# Untapped research opportunities in China: promising future avenues and potential concerns for aging companion animals

**DOI:** 10.1007/s11357-021-00433-y

**Published:** 2021-08-24

**Authors:** Jessica M. Hoffman, Shanshan Song, Katharina Brugger, Teresa G. Valencak

**Affiliations:** 1 Department of Biology, University of Alabama At Birmingham, Birmingham, AL 35294 USA; 2 3000 Animal Hospital Center, 103 T5 EFC, Cangqian Street, Yuhang District, Hangzhou, Zhejiang China; 3 Unit for Veterinary Public Health and Epidemiology, University of Veterinary Medicine Vienna, Veterinärplatz 1, 1210 Vienna, Austria; 4 College of Animal Sciences, Zhejiang University, Hangzhou, China; 5 Department of Biosciences, Paris Lodron University Salzburg, Salzburg, Austria

**Keywords:** Companion animals, China, Aging research, Animal welfare, Economy

## Abstract

Companion animals have recently been proposed as ideal translational models of human aging due to their shared susceptibility for certain diseases, similar environments, and sophisticated veterinary medicine diagnostics, all of which are not possible in rodent laboratory models. Here, we introduce and propose the study of companion animals in China as a largely untapped resource in academic and veterinary aging research. Pet ownership rates along with economic gains in the pet industry have skyrocketed over the last decade in China. Yet, the majority of research institutions still focus on agricultural animal research, not companion animals. In this perspective, we compare available pet ownership rates between the USA, the European Union, and China before focusing on the potential of companion animal aging research in China. In addition, we highlight some ethical considerations that must be addressed before large-scale companion animal aging research can be completed.

Over the past century, companion animals, most often cats and dogs, have made a transition from “property” to “family” in industrialized nations. Instead of keeping pets outside for working purposes, e.g., guarding, herding, and pest reduction, domesticated pets have moved into the home where their most common use is companionship. Along with the rise in pets as family members, there has been a significant increase in the veterinary care; individuals are willing to give their pets, and with better veterinary care comes improved health and longer lifespans. Pets today are living longer, healthier lives than ever before. This is not just due to shifts in their environment, but the significant monetary resources owners will put towards their pets’ health [1].

While the treatment of aging pets has now been a significant issue of consideration for companion animal veterinarians in the West, it has only recently become an issue in many other countries, including China. In China, the rates of dog ownership have more than doubled in just 10 years (Fig. 1A), and cat ownership numbers have quadrupled during this time, with no signs of slowing down. This increase is also associated with changes in the environments of companion animals, as dogs and cats are now living inside their owners’ homes, as compared to strictly outdoors [2]. Rapid increases in pet ownership began in 1992 when China removed a ban on pets in urban settings [1], driving unprecedented growth in the numbers of companion cats and dogs in large cities (Fig. 1B, [3]). This ban was put in place by the Chinese Communist Party in an attempt to eradicate many zoonotic diseases as well as allow for “normal” activities of daily living in the city [3], and to this day, there are still many rules in specific cities with regards to companion pet ownership. With the shift in policy, there has also been a shift in the perception of cats and dogs in China, similar to what was seen in the USA, Canada, and Europe several decades before, and younger Chinese have a much more positive attitude towards animals than older adults [4]. However, with this significant rise in pet ownership in only 30 years, some serious considerations must be addressed. Here, we provide insights into the potential of China to become a future area of pet aging research while also pointing out some ethical issues.

**Fig. 1:**
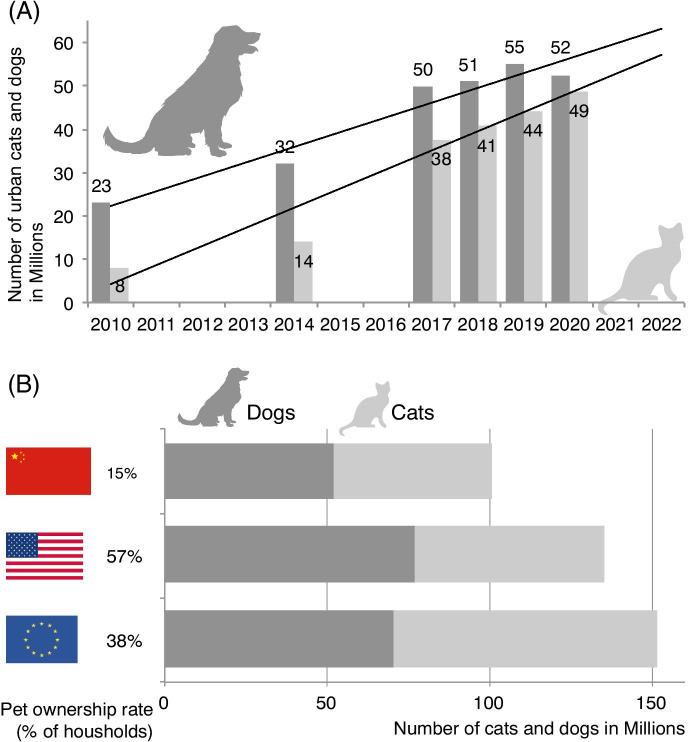
**A** Time courses of urban cats and dogs in Chinese urban households between 2010 and 2022 (compiled from [5–9]). **B** Number of cats and dogs in China, the EU, and the USA as well as overall pet ownership rates (compiled from [8, 9])

We are writing this perspective with the purpose to bring forth two large ideas about companion pet aging in China: (1) the increases in Chinese pet ownership provide a largely untapped resource for aging research, especially if the country can move towards more companion animal research as compared to agricultural research, and (2) as pet ownership increases and pets age, there will be a need for more general practice veterinarians, veterinary technicians, and veterinary specialists, as well as increased pet dietary guidelines.

More individuals are buying/adopting pets for companionship in China than ever before, and many of these animals are receiving care similar to their counterparts in the USA, Canada, and Europe. Not surprisingly, this has led to an increase in pet clinics and grooming salons across China (https://www.chinapetmarket.com/articles/trends/, [5–8]). In addition, very recently investment firms have placed large amounts of capital in pet-related industries across the globe [7], and this can also be seen in China [10]; PriceWaterCoopers, 2020 https://www.pwccn.com/en]. China is at the forefront of development of “smart” devices for pets as well as the application of artificial intelligence in the pet product sector, and Chinese individuals tend to be more open and accepting of artificial intelligence than other Western countries [12]. There has also been a push to use artificial intelligence as a means of developing aging and longevity interventions [5–8, 11] that presumably could also be applied to companion animals. As the development of smart pet products provides insights into pet physical health, we may gain more information from Chinese pet owners than others in the West.

Companion animals, especially the companion dog, have recently been proposed as strong models of human aging and age-related diseases [13–15]. This is due to multiple factors including the development of similar morbidities, the high levels of veterinary diagnostics and treatments available, and the fact that they live in the same environment as their owners. Thus, companion animals tend to reflect their human owners in a way that is not possible in laboratory settings. This has led to the development of prospective studies on canine aging, as shown by the recently started Dog Aging Project in the USA [16]. While companion animals have just begun to become popular in China, they already show some of the expected characteristics of pets in the Western world. For example, companion dogs in China show high rates of obesity at all ages [17], which may be a reflection of the increasing levels of obesity in the Chinese population [18]. Therefore, companion animals are poised to reflect many of the clinical and aging phenotypes seen in their owners in China, similar to what is observed in the USA, Canada, and EU. In addition, research has shown that similar to other countries, companion dogs can provide companionship and improve mental health in Chinese older adults [19], and pet ownership in China is associated with a decreased risk of cardiovascular disease [20]. These combined suggest companion animals may provide a large untapped research avenue for both the biomedical, epidemiological, and social sciences in China.

While companion animal resources are growing dramatically in China, peer-reviewed academic research on companion animal aging is sparse, as the majority of research is conducted on agricultural animals. However, there are apparent trends in companion animal aging that we hope will soon be more thoroughly researched. Anecdotally, there have been significant shifts in the demographics of pets brought into veterinary clinics. One of our authors, Dr. Shanshan Song, has noticed a significant increase in the age and overall longevity of companion animals she is treating daily in primary veterinary care. This is at least partially driven by a decrease in pets presenting with infectious diseases, due to increases in preventative vaccinations. Zoonotic infections were the main source of morbidity in companion animals in China previously, and the majority of companion dog and cat research deals specifically with prevalence of infectious diseases [21]. However, today, primary care clinics are seeing significant increases in metabolic, immune-mediated, and neoplasia disorders that are commonly seen in older, aging pets. These increases in age-related disease are accompanied with changes in owner perceptions of longevity. Just 10 years ago, many owners believed their pet would not live past 7 or 8 years, and now, most expect their pets to live well into their mid-teens. All combined these suggest that, in the last decade, Chinese owners are putting significant resources into the health of their pets, and collaborations with aging researchers could prove very fruitful. However, peer-reviewed companion animal aging research out of China is still in its infancy.

One of the major facets of research we see companion animals being especially useful is in the field of environmental impacts, health, and aging. The majority of pet owners in China live in large cities, where concurrently, the air is often of the lowest quality [22]. In the last couple of years, many epidemiological studies have begun to look at the role of poor air quality on health and lifespan in humans living in megacities [e.g. 23. Therefore, like their owners, these cats and dogs are exposed daily to many airborne pollutants and small particles. On the other hand, many pets in China live their lives completely indoors where air filters and cleaning robots improve the indoor living climate. Potentially, there could be interesting hypotheses tested on the rates of aging between those animals that have high versus low exposure to environmental variations and pollution. As companion animals age much faster than their human counterparts, we can begin to understand the molecular and physiological changes that occur in response to chronic pollution exposure in pets in a shorter time frame than longitudinal studies in humans.

In addition to the adoption of many veterinary techniques of the West, Chinese veterinary medicine also employs traditional Eastern practices which could be of interest to aging research. One specific area with promise is the use of veterinary acupuncture. Acupuncture has been practiced in China for over 3000 years [24] and has shown tremendous growth recently in Western countries. Within the areas of pain management and cardiovascular parameters, acupuncture has been used in companion animals in both the East and West; however, rigorous clinical trials are rare, making it difficult so far to draw conclusions on the effects of acupuncture [25]. However, acupuncture has the potential to be a therapy to alleviate symptoms of many age-related disorders in companion pets. Therefore, Chinese traditional medicine may also have some clinical promises in aging pets, but it needs to be addressed with more rigorous peer-reviewed research.

While the use of companion pets in aging research in China is an interesting idea, there are still many ethical considerations that must be attended to before moving into large-scale research. Many individuals in China still think of pets more for their usefulness and economic benefit to humans instead of as companions, a utilitarian viewpoint of animal ownership [26]. With this prevailing idea, there is a potential for exploitation of these pets. To help address some of these issues, there has been a push in Chinese veterinary education to think more about animal welfare. This will hopefully move into the general public as many Chinese individuals do not know the meaning of “animal welfare” [27]. If pets are still thought of in a utilitarian way, animal welfare is less likely to be considered, and the ideal treatment of age-related diseases may not occur, as owners (and researchers) may not want to put the same resources as would be seen in other countries. We think there is a need both among veterinarians and the general population in China to understand the nuances of animal welfare and to embrace the many different treatments for age-related morbidities that are consistently prescribed in the West. This will entail a shift in perception of pets and will require the investment of monetary resources from owners that may not be expected yet. However, we must note that, as described above, it appears at least younger adults in large cities have already made this mental shift.

Overall, we believe that China is well poised to become a leader in companion animal aging research in the coming decades. While they are almost a century behind Western countries in companion pet research, the rapidly growing cat and dog population, coupled with the shift to view animals as companions, has the potential for very fruitful research in China and with other countries [28]. We hope to see research networks develop between researchers, veterinarians, and industry in ways that all groups profit from. We are excited to see where China moves in this realm, towards becoming a top country for companion animal ownership.

## Data Availability

Not applicable.
